# Achievable Rate Estimation of IEEE 802.11ad Visual Big-Data Uplink Access in Cloud-Enabled Surveillance Applications

**DOI:** 10.1371/journal.pone.0167447

**Published:** 2016-12-20

**Authors:** Joongheon Kim, Jong-Kook Kim

**Affiliations:** 1 School of Computer Science and Engineering, Chung-Ang University, Seoul 069-74, Republic of Korea; 2 School of Electrical Engineering, Korea University, Seoul, 136-713, Republic of Korea; West Virginia University, UNITED STATES

## Abstract

This paper addresses the computation procedures for estimating the impact of interference in 60 GHz IEEE 802.11ad uplink access in order to construct visual big-data database from randomly deployed surveillance camera sensing devices. The acquired large-scale massive visual information from surveillance camera devices will be used for organizing big-data database, i.e., this estimation is essential for constructing centralized cloud-enabled surveillance database. This performance estimation study captures interference impacts on the target cloud access points from multiple interference components generated by the 60 GHz wireless transmissions from nearby surveillance camera devices to their associated cloud access points. With this uplink interference scenario, the interference impacts on the main wireless transmission from a target surveillance camera device to its associated target cloud access point with a number of settings are measured and estimated under the consideration of 60 GHz radiation characteristics and antenna radiation pattern models.

## 1 Introduction

As actively discussed and studied in, centralized cloud-enabled network architecture which is interacting with randomly deployed wireless and mobile users such as Internet-of-Things (IoT) devices have received a lot of attention by industry and academia research groups.

In cloud-enabled networks, the data within the cloud storage can be obtained from Internet (via wireline Ethernet connections) or from randomly deployed wireless users (via wireless access points). On top of the concept of crowdsourcing [1–9], this paper considers a situation where the organized and structured big-data within the cloud storage is obtained from the deployed wireless users. In large-scale wireless users, the simultaneous uplink transmission toward their associated access points can generate interference among the simultaneously activated wireless links. Therefore, the performance evaluation of the simultaneous uplink wireless transmission is important for designing this cloud storage centric wireless architectures.

In this given reference network architecture, one of the most challenging research topics related to the uplink wireless transmission is simultaneous large-scale data sharing under the consideration of interference existence. For large-scale and high-rate wireless transmission, millimeter-wave wireless communication technologies are widely discussed nowadays [10–13]. In millimeter-wave wireless communication research, 60 GHz wireless technologies are generally and widely considered [14] because it is only one standardized millimeter-wave wireless technology so called IEEE 802.11ad [15].

In this paper, this 60 GHz wireless channel is considered for simultaneous large-scale big-data information acquisition based on following reasons:

- **Multi-gigabit-per-second data rates:** The currently existing millimeter-wave wireless schemes are able to support multi-gigabit per second (Gbps) data rates because of their ultra-wide-band channel bandwidth. In this given cloud-based wireless network architectures, supporting high data rates is important for large-scale big-data storage construction in the centralized cloud platforms.
- **High directionality:** Millimeter-wave wireless transmission is extremely high directional due to its high carrier frequencies (from 30 GHz to 300 GHz). According to the fact that large-scale and densely deployed wireless uses will perform uplink data transmission, high interference generation is expected. If the transmission beams are quite narrow (i.e., high directional), the interference impacts will be reduced. Therefore, the high directionality of millimeter-wave propagation is helpful in terms of interference reduction.

Therefore, this paper calculates achievable rates in one main link (from a target wireless user to its associated target access point) when interference exists (i.e., from interfering wireless users to the target access point while the transmit antennas of interfering wireless users are aligned with their associated interfering access points) in the 60 GHz band.

**Fig 1:**
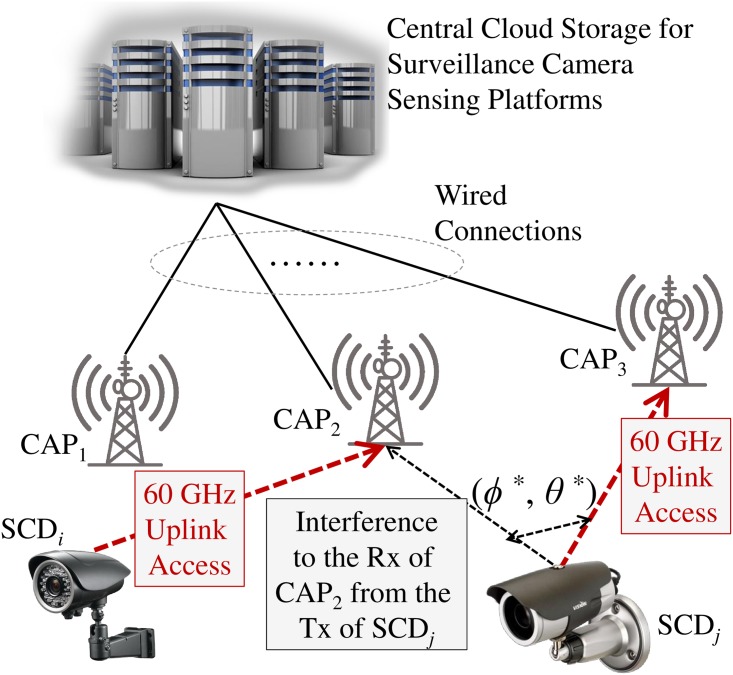
A reference 60 GHz visual big-data uplink access in cloud-enabled camera-sensing platforms where CAP and SCD stand for a cloud-enabled access point and a surveillance camera device, respectively.

In this paper, we discuss about specific *visual* big-data uploading case for cloud-enabled surveillance applications (detailed architectural descriptions are in Sec. 2.2 and illustrated in Fig 1). For uploading high volume visual big-data, we especially require high-rate wireless technologies, therefore, 60 GHz millimeter-wave is only one option we can choose. Moreover, IEEE 802.11ad [15] is originally designed for wireless high definition video streaming.

This analysis is essential to justify the use of 60 GHz in cloud-enabled wireless networks. If the level of generated interference is inadmissibly high, it negatively impacts the link for cloud radio access, which would discourage users from adopting 60 GHz based cloud wireless networks over the traditional concepts. Thus, this research is worthy in addressing this essential problem, and is considered as the first step for the understanding of 60 GHz uplink access for simultaneous large-scale data uploading to cloud storage.

The last sections of this paper are as follows: The preliminary knowledge of uplink interference estimation in 60 GHz IEEE 802.11ad wireless access is presented in Sec. 2. The achievable rate computation in uplink 60 GHz wireless access is explained in Sec. 3 with 60 GHz propagation characteristics when interference exists. The related intensive simulation results are presented in Sec. 4 and Sec. 5 concludes this paper.

## 2 Preliminary Knowledge

This section addresses related work (see Sec. 2.1), reference network models (see Sec. 2.2), and 60 GHz radiation characteristics including path-loss, oxygen attenuation, and antenna models (see Sec. 2.3), respectively.

### 2.1 Related Work

The probability of interference occurrence in directional millimeter-wave wireless communications is $\left(\frac{\phi_{\text{bw}}}{360^{{}^{\circ}}}\cdot \frac{\theta_{\text{bw}}}{360^{{}^{\circ}}}\right)$ where *ϕ*_bw_ and *θ*_bw_ are beamwidth values in azimuth and elevation planes (i.e., 0° ≤ *ϕ*_bw_ ≤ 360°, 0° ≤ *θ*_bw_ ≤ 360°), as theoretically analyzed in [16]. According to the fact that the beamwidth of millimeter-wave wireless communications is generally between 1° and 10° [10], one of major believes among millimeter-wave wireless communication engineers is interference impacts are ignorable in millimeter-wave wireless system design and implementation.

Despite this theoretical analysis result, massive accumulation of millimeter-wave interference components may not be ignorable as numerically studied in [17, 18].

As well presented in [17], 60 GHz IEEE 802.15.3c millimeter-wave propagation and its interference impacts were studied in two-tiered camera-sensing wireless networks. However, the proposed numerical approach in [17] only considers 2D antenna radiation patterns, i.e., only an azimuth plane is considered and an elevation plane is considered to be omni-directional which is not true in highly directional millimeter-wave radio propagation [19].

The presented research approach in [18] is different from the approach in this paper because the simulation study in [18] considers 38 GHz millimeter-wave radio propagation (not 60 GHz). In addition, the considering antenna radiation pattern is no equivalent to the patterns in this paper because the patterns in [18] considers the standardized International Telecommunication Union (ITU) 1336 model [20] whereas this paper considers theoretical steerable antenna models with Gaussian mainlobe profile. Even though the ITU 1336 model is also widely used in the literatures, considering this steerable model with Gaussian mainlobe profile is more reasonable because steerable antennas are currently actively used in realistic millimeter-wave cellular and access network research [13, 21–23].

Moreover, the research results in [17, 18] calculate estimated achievable rates with Shannon capacity equation which holds only optimum modulation and coding schemes are assumed. In addition to this Shannon capacity equation-based achievable rate calculation, this paper additionally considers achievable rate estimation with practical modulation and coding scheme (MCS) formats defined in IEEE 802.11ad specification [15] (more details will be presented in Sec. 3.2).

### 2.2 Reference 60 GHz Visual Big-Data Uplink Access in Cloud-Enabled Surveillance Applications

The reference platform and big-data storage architecture with 60 GHz cloud access points (CAPs) is illustrated in Fig 1. To enable visual big-data information processing in centralized storage for surveillance applications, the first step is collecting corresponding massive visual data. With the concept of crowdsourcing [7], the visual and image data can be gathered from surveillance camera devices (SCDs) via densely deployed CAPs to cloud storage as illustrated in Fig 1 [24–26].

The uplink wireless access from SCDs to CAPs are using a 60 GHz millimeter-wave channel because it supports (i) high speed data rates with ultrawidebandwidth channel which is suitable for massive visual big-data information uploading; and (ii) directional transmission which can avoid interference among ultra-densely deployed 60 GHz surveillance devices.

In the Fig 1, one example of interference impacts is illustrated. For the 60 GHz uplink wireless transmission from SCD_*i*_ to CAP_2_, one interference source exists, i.e., 60 GHz uplink wireless transmission from SCD_*j*_ to CAP_3_. Therefore, the transmission from SCD_*j*_ to its associated CAP_3_ is interfering the transmission from SCD_*i*_ to its associated CAP_2_ at CAP_2_.

If a lot of SCDs are performing uplink wireless access simultaneously, the accumulated interference at the interesting receiver (i.e., CAP_2_ in Fig 1) should be calculated to observe the impacts in terms of capacity degradation. This simulation study is about to observe this degradation and observes the actual capacity reduction impacts due to the accumulated interference impacts. The capacity will be calculated with two ways, i.e., (i) Shannon capacity equation based estimation and (ii) IEEE 802.11ad MCS based achievable rate estimation.

### 2.3 Radio Propagation Characteristics at 60 GHz Channels

This section explains radiation characteristics in 60 GHz millimeter-wave radio propagation including path-loss, oxygen attenuation, and steerable antenna radiation patterns with Gaussian mainlobe profiles.

#### 2.3.1 Path-loss at 60 GHz channels

The 60 GHz path-loss model in the IEEE 802.11ad standards for line-of-sight (LoS) scenarios is as follows [7]:

$$L\left(d\right)=68.0630+20\log_{10}\left(d\right)$$ (1)

where *d* denotes a distance a CAP and a SCD (in a meter scale). This paper assumes that there is no blockage between cloud access points and surveillance devices, therefore only a 60 GHz LoS path-loss model is considered.

#### 2.3.2 Oxygen attenuation at 60 GHz channels

The oxygen attenuation *O*(*d*) is observed as 16 dB/Km [16], i.e., $O\left(d\right)=16\cdot \frac{d}{1000}$ where *d* denotes a distance a CAP and a SCD (in a meter scale).

#### 2.3.3 Reference steerable antenna radiation patterns with Gaussian mainlobe profile

The reference steerable antenna model in this paper contains a mainlobe with Gaussian profile. The mainlobe can be formulated as follows using two-dimensional Gaussian function:

$$G_{\text{mW}}\left(\phi ,\theta \right)=G_{\text{mW}}^{\max}\cdot \exp\left(-\gamma^{\phi }\cdot \phi^{2}\right)\cdot \exp\left(-\gamma^{\theta }\cdot \theta^{2}\right)$$ (2)

where *γ*^*ϕ*^ and *γ*^*θ*^ are the values determined by the beamwidths in azimuth and elevation planes when the beamwidth is defined as the angle where the statistical power distribution is the half of peak. Thus it is also called to half-power beamwidth (HPBW). Therefore,

$$\frac{G_{\text{mW}}\left(\phi ,\theta \right)}{G_{\text{mW}}^{\max}}=\exp\left\{-\gamma^{\phi }\cdot {\left(\frac{\phi_{\text{bw}}}{2}\right)}^{2}\right\}=0.5,$$ (3)

$$\frac{G_{\text{mW}}\left(\phi ,\theta \right)}{G_{\text{mW}}^{\max}}=\exp\left\{-\gamma^{\theta }\cdot {\left(\frac{\theta_{\text{bw}}}{2}\right)}^{2}\right\}=0.5,$$ (4)

where *ϕ*_bw_ and *θ*_bw_ are beamwidth values in azimuth and elevation planes; and then *γ*^*ϕ*^ and *γ*^*θ*^ can be expressed as follows:

$$\gamma^{\phi }=\frac{4\ln2}{\phi_{\text{bw}}^{2}}\text{ and }\gamma^{\theta }=\frac{4\ln2}{\theta_{\text{bw}}^{2}}.$$ (5)

and thus *G*_dBi_(*ϕ*, *θ*), i.e., the dBi scale value of *G*_mW_(*ϕ*, *θ*) in Eq (2), can be presented as follows with Eq (5):

$$G_{\text{dBi}}\left(\phi ,\theta \right)=G_{\text{dBi}}^{\max}+10\cdot \log_{10}\left[\exp\left\{-4\ln2\cdot {\left(\frac{\phi }{\phi_{\text{bw}}}\right)}^{2}\right\}\right]+10\cdot \log_{10}\left[\exp\left\{-4\ln2\cdot {\left(\frac{\theta }{\theta_{\text{bw}}}\right)}^{2}\right\}\right],$$ (6)

due to

$$G_{\text{dBi}}\left(\phi ,\theta \right)=10\cdot \log_{10}\left\{G\left(\phi ,\theta \right)\right\},$$ (7)

when $G_{\text{dBi}}^{\max}$ is defined as the dB scale of $G_{\text{mW}}^{\max}$, and eventually,

$$G_{\text{dBi}}\left(\phi ,\theta \right)\approx G_{\text{dBi}}^{\max}-12{\left(\frac{\phi }{\phi_{\text{bw}}}\right)}^{2}-12{\left(\frac{\theta }{\theta_{\text{bw}}}\right)}^{2}.$$ (8)

The *ϕ*_bw_ and *θ*_bw_ in Eq (8) can be calculated as follows [27]:

$$\theta_{\text{bw}}=\frac{31000\cdot 10^{-\frac{G_{\text{dBi}}^{\max}}{10}}}{\phi_{\text{bw}}}$$ (9)

and we assume *θ*_bw_ ≈ *ϕ*_bw_, therefore

$$\phi_{\text{bw}}\approx \theta_{\text{bw}}\approx {\left(31000\cdot 10^{-\frac{G_{\text{dBi}}^{\max}}{10}}\right)}^{\frac{1}{2}}$$ (10)

by Eq (9). Note that we assume $G_{\text{dBi}}^{\max}=24$ dBi.

**Fig 2:**
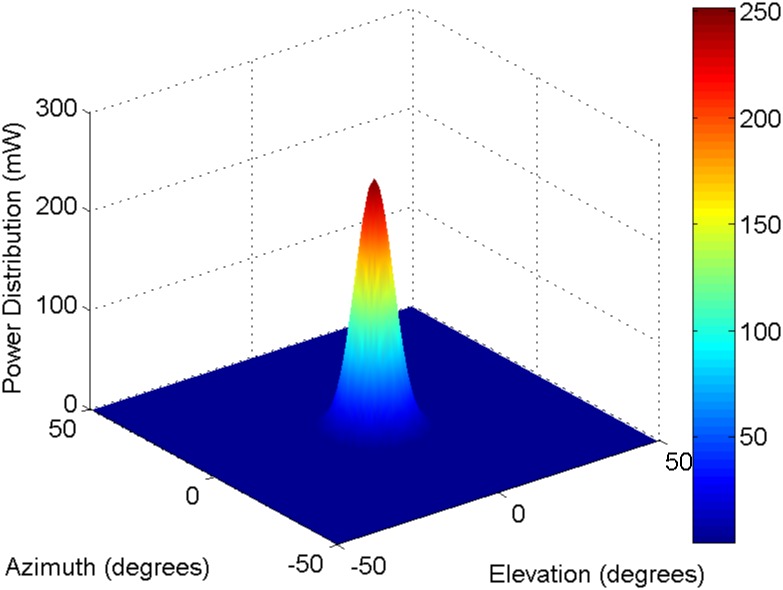
Reference steerable antenna radiation patterns with Gaussian mainlobe profile with the maximum antenna gain of 24 dBi (in a linear scale).

Based on this reference steerable antenna model with Gaussian mainlobe profiles, three-dimensional antenna radiation patterns (in a linear scale) are as plotted in Fig 2.

#### 2.3.4 Discussion

As presented in the measurement results of the Millimetre-Wave Evolution for Backhaul and Access (MiWEBA) project [28, 29], multi-path and small-scaling fading impacts are not seriously considered in 60 GHz millimeter-wave outdoor backhaul and access networking scenarios. According to the fact that our visual big-data uplink access systems will be generally deployed in outdoor applications, we did not take account the impact in this contribution.

## 3 Achievable Rate Estimation

In order to do the achievable rate estimation, this paper considers two approaches, i.e., (i) achievable rate estimation with Shannon capacity equation (refer to Sec. 3.1) and (ii) achievable rate estimation with IEEE 802.11ad MCS (refer to Sec. 3.2), respectively. This section discusses more details about the two approaches as following two subsections.

### 3.1 Achievable Rate Calculation with Shannon Equation

To estimate achievable data rates, Shannon’s equation can be used, i.e.,

$$R_{\ell }\left(d\right)={\text{BW}}_{60\text{GHz}}\cdot \log_{2}\left(1+\frac{P_{\text{mW}}^{\ell ,\text{RX}}\left(d\right)}{n_{\text{mW}}+\sum_{i\in L,i\neq \ell }\;I_{\text{mW}}^{i,\ell }}\right)$$ (11)

where *R*_*ℓ*_(*d*) is an achievable data rate in wireless link *ℓ* where *d* is the distance between the transmitter and receiver of the link *ℓ*, BW_60GHz_ is the channel bandwidth in a 60 CHz channel (i.e., 2.16 GHz [10, 11]), $P_{\text{mW}}^{\ell ,\text{RX}}\left(d\right)$ is the received signal power at the receiver of link *ℓ* in a mW (i.e., 10^−3^ Watt) scale, $I_{\text{mW}}^{i,\ell }$ is an interference to the receiver of link *ℓ* from the transmitter of link *i* in a mW scale, *n*_mW_ is a background noise in a mW scale, and L is the set of all given links, respectively.

The detailed computation procedures of $P_{\text{mW}}^{\ell ,\text{RX}}\left(d\right)$, $I_{\text{mW}}^{i,\ell }$, and *n*_mW_ are explained in following Sec. 3.1.1, Sec. 3.1.2, and Sec. 3.1.3, respectively.

#### 3.1.1 Received signal strength at the receiver of main link *ℓ*

The received signal strength at the receiver of link *ℓ*, i.e., $P_{\text{mW}}^{\ell ,\text{RX}}\left(d\right)$ in a mW scale, can be calculated as follows:

$$P_{\text{mW}}^{\ell ,\text{RX}}\left(d\right)=10^{\left(\frac{P_{\text{dBm}}^{\ell ,\text{RX}}\left(d\right)}{10}\right)}$$ (12)

$$=10^{\left(\frac{G_{\text{dBi}}^{\ell ,\text{TX}}+P_{\text{dBm}}^{\ell ,\text{TX}}-L^{*}\left(d\right)+G_{\text{dBi}}^{\ell ,\text{RX}}}{10}\right)}$$ (13)

where $G_{\text{dBi}}^{\ell ,\text{TX}}$ is a transmit antenna gain at the transmitter of link *ℓ*, $P_{\text{dBm}}^{\ell ,\text{TX}}$ is a transmit power at the transmitter of link *ℓ*, L*(*d*) is attenuation depending on *d* which can be calculated as L*(*d*) = *L*(*d*) + *O*(*d*) where *L*(*d*) is path-loss depending on *d* (refer to Sec. 2.3.1), *O*(*d*) is oxygen attenuation depending on *d* (refer to Sec. 2.3.2), and $G_{\text{dBi}}^{\ell ,\text{RX}}$ is a receive antenna gain at the receiver of link *ℓ*, respectively. As explained in [10, 11], the equivalent isotropically radiated power (EIRP) is 43 dBm in USA. Therefore, we assume $G_{\text{dBi}}^{\ell ,\text{TX}}+P_{\text{dBm}}^{\ell ,\text{TX}}=19+24=43$ dBm. In addition, $G_{\text{dBi}}^{\ell ,\text{RX}}$ is assumed to be 0 dBi, i.e., the receiver antennas are omni-directional.

#### 3.1.2 Interference estimation from the transmitter of link *i* to the receiver of main link *ℓ*

In Eq (11), $I_{\text{mW}}^{i,\ell }$ stands for the interference in a mW scale to the receiver of link *ℓ* from the transmitter of link *i* where i≠ℓ,i∈L [18]. This is illustrated in Fig 1. Therefore, the interference from the transmitter of link *i* transmission to the receiver of link *k* can be as follows:

$$I_{\text{mW}}^{i,\ell }=10^{\left(\frac{I_{\text{dBm}}^{i,\ell }}{10}\right)}$$ (14)

$$=10^{\left(\frac{G_{\text{dBi}}^{i,\text{TX}}\left(\phi^{*},\theta^{*}\right)+P_{\text{dBm}}^{i,\text{TX}}-L^{*}\left(d_{\left(i,\ell \right)}\right)+G_{\text{dBi}}^{\ell ,\text{RX}}}{10}\right)}$$ (15)

where $P_{\text{dBm}}^{i,\text{TX}}$ is the transmit power from the transmitter of link *i*; and L* (*d*_(*i*,*ℓ*)_) is attenuation depending on the distance between the transmitter of link *i* and the receiver of link *ℓ*, i.e., *d*_(*i*,*ℓ*)_, where this L* (*d*_(*i*,*ℓ*)_) can be calculated with L* (*d*_(*i*,*ℓ*)_) = *L* (*d*_(*i*,*ℓ*)_) + *O* (*d*_(*i*,*ℓ*)_) where *L* (*d*_(*i*,*ℓ*)_) is path-loss attenuation depending on *d*_(*i*,*ℓ*)_, *O* (*d*_(*i*,*ℓ*)_) is oxygen attenuation depending on *d*_(*i*,*ℓ*)_, and $G_{\text{dBi}}^{i,\text{TX}}\;(\phi^{*},\theta^{*})$ is an antenna radiation gain from the transmitter of link *i* to the receiver of link *ℓ* while the transmit antenna of the transmitter of link *i* is aligned with the receiver of link *i*, i.e., the aligned 60 GHz radio wave propagation that is generated by the transmit antenna of the transmitter of link *i*. Therefore, ϕ* and θ* are the angular differences between the transmitter of link *i* and the receiver of link *ℓ* while the transmitter of link *i* is aligned to the receiver of link *i*. Note that $G_{\text{dBi}}^{\ell ,\text{Rx}}$ is assumed to be 0 dBi, in this interference estimation procedure because the receiver antennas are assumed to be omni-directional.

#### 3.1.3 Background noise

In Eq (11), *n*_mW_ is a background noise that can be calculated as

$$n_{\text{mW}}=10^{\left(\frac{n_{\text{dBm}}}{10}\right)}$$ (16)

$$=10^{\left(\frac{k_{B}T_{e}+10\log_{10}\left({\text{BW}}_{60\text{GHz}}\right)+L_{\text{implementation}}+F_{N}}{10}\right)}$$ (17)

due to [30] where *n*_mW_ is a background noise in a mW scale, *n*_dBm_ is a background noise in a dBm scale, *k*_*B*_*T*_*e*_ is a noise power spectral density that is *k*_*B*_*T*_*e*_ = −174 dBm/Hz [30], BW_60GHz_ is a channel bandwidth (i.e., 2.16 GHz at 60GHz [10, 11]), *L*_implementation_ is implementation loss and this is assumed to be 10 dB [15], and *F*_*N*_ is a noise figure and this is assumed to be 5 dB [15], respectively. Therefore, *n*_dBm_ = −65.6555 dBm and thus

$$n_{\text{mW}}=2.7193\times 10^{-7}\text{mW}\;.$$ (18)

### 3.2 Achievable Rate Calculation with IEEE 802.11ad MCS

The most well-known approach to calculate physical data rates is using the Shannon’s capacity equation (refer to Sec. 3.1). However, this Shannon’s equation holds only optimum modulation and coding schemes are considered. Thus, more practical approach is additionally required. The practical rate calculation procedure is as follows:

- Calculating received signal strength at a receiver from a transmitter depending on distance (details are in Section 3.2.1);
- Comparing signal strength with receiver sensitivity values associated with the set of MCS defined in [15]. On this basis, the supportable MCSs and their associated achievable rates can be found (details are in Section 3.2.2).

#### 3.2.1 Step #1: Calculating received signal strength at a receiver

The received signal strength at the receiver of main link *ℓ* in a dBm scale can be computed as follows using Eq (13):

$$P_{\text{dBm}}^{\ell ,\text{RX}}\left(d\right)=G_{\text{dBi}}^{\ell ,\text{TX}}+P_{\text{dBm}}^{\ell ,\text{TX}}-L^{*}\left(d\right)+G_{\text{dBi}}^{\ell ,\text{RX}}.$$ (19)

#### 3.2.2 Step #2: Comparing signal strength with receiver sensitivity values associated with the set of MCS

In this second step, the supportable MCS can be found using the IEEE 802.11ad based on the calculated received signal strength using Eq (19) in Sec. 3.2.1. This is done by comparing the calculated values with receiver sensitivity values defined in the IEEE 802.11ad Table 21-3 (presented in Tables 1 and 2) [15]. For example, if the calculated received signal strength is −61.5 dBm, the supportable MCS is MCS7 and its associated achievable rate is 1925 Mbps.

**Table 1:** IEEE 802.11ad Rx Sensitivity Table [15].

Rx Sensitivity	Supportable MCS	Achievable Rates
−78 dBm	MCS0	27.5 Mbps
−68 dBm	MCS1	385 Mbps
−66 dBm	MCS2	770 Mbps
−65 dBm	MCS3	962.5 Mbps
−64 dBm	MCS4	1155 Mbps
−63 dBm	MCS6	1540 Mbps
−62 dBm	MCS7	1925 Mbps
−61 dBm	MCS8	2310 Mbps
−59 dBm	MCS9	2502.5 Mbps
−55 dBm	MCS10	3080 Mbps
−54 dBm	MCS11	3850 Mbps
−53 dBm	MCS12	4620 Mbps

**Table 2:** Relationship between Shannon Capacity and MCS Levels.

Shannon Capacity	Supportable MCS
0.1430 × 10^10^	MCS0
0.2039 × 10^10^	MCS1
0.2403 × 10^10^	MCS2
0.2809 × 10^10^	MCS3
0.3254 × 10^10^	MCS4
0.3739 × 10^10^	MCS6
0.4253 × 10^10^	MCS7
0.5380 × 10^10^	MCS8
0.7902 × 10^10^	MCS9
0.8565 × 10^10^	MCS10
0.9231 × 10^10^	MCS11
4.3477 × 10^10^	MCS12

### 3.3 Comparison of Two Different Achievable Rate Calculation Procedures

**Fig 3:**
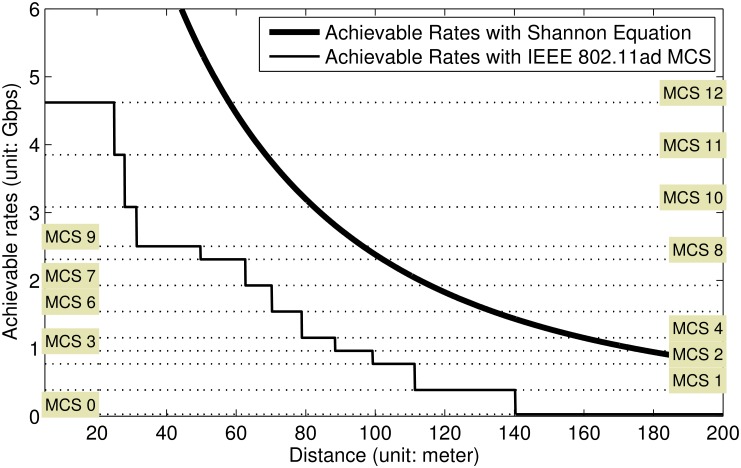
Achievable rate calculation with Shannon equation and IEEE 802.11ad MCS.

The achievable rate calculated with Shannon capacity equation is as follows:

$$R_{\ell }\left(d\right)={\text{BW}}_{60\text{GHz}}\cdot \log_{2}\left(1+\frac{P_{\text{mW}}^{\ell ,\text{RX}}\left(d\right)}{n_{\text{mW}}}\right)$$ (20)

under the assumption that there are no interference components. This is plotted in Fig 3. With the calculated $P_{\text{mW}}^{\ell ,\text{RX}}\left(d\right)$, MCS-based achievable rate computation results are also presented in Fig 3. Since Shannon capacity equation holds only when optimum modulation and coding schemes are assumed, MCS-based rate computation results are always lower than the plotting derived from Shannon capacity equation.

Based on Fig 3, we can observe the relationship between achievable rates (with Shannon equation) and MCS levels. For example, if current calculated Shannon capacity value is 0.5000 × 10^10^, MCS7 is the maximum among supportable MCS levels, as plotted in Fig 3 and also presented in Table 2.

For the cases where interference sources exist, we can derive supportable MCS and its corresponding achievable rate can be derived as follows: After calculating Eq (11) with calculated interference impacts, we can match the calculated achievable rate using Eq (11) with the Table 2. Then, we can finally find supportable MCS and its corresponding achievable rate under the situation that there exists interference components. In addition, the practical hardware prototype with IEEE 802.11ad can suffer from additional communication overheads. However, the impacts depend on the system, i.e., those are not considered in this contribution.

## 4 Performance Estimation

This section provides large-scale intensive numerical simulation results with the given 60 GHz radio propagation characteristics in Sec. 2.3 and the two computational procedures for calculating achievable rates in Sec. 3.1 and Sec. 3.2.

**Fig 4:**
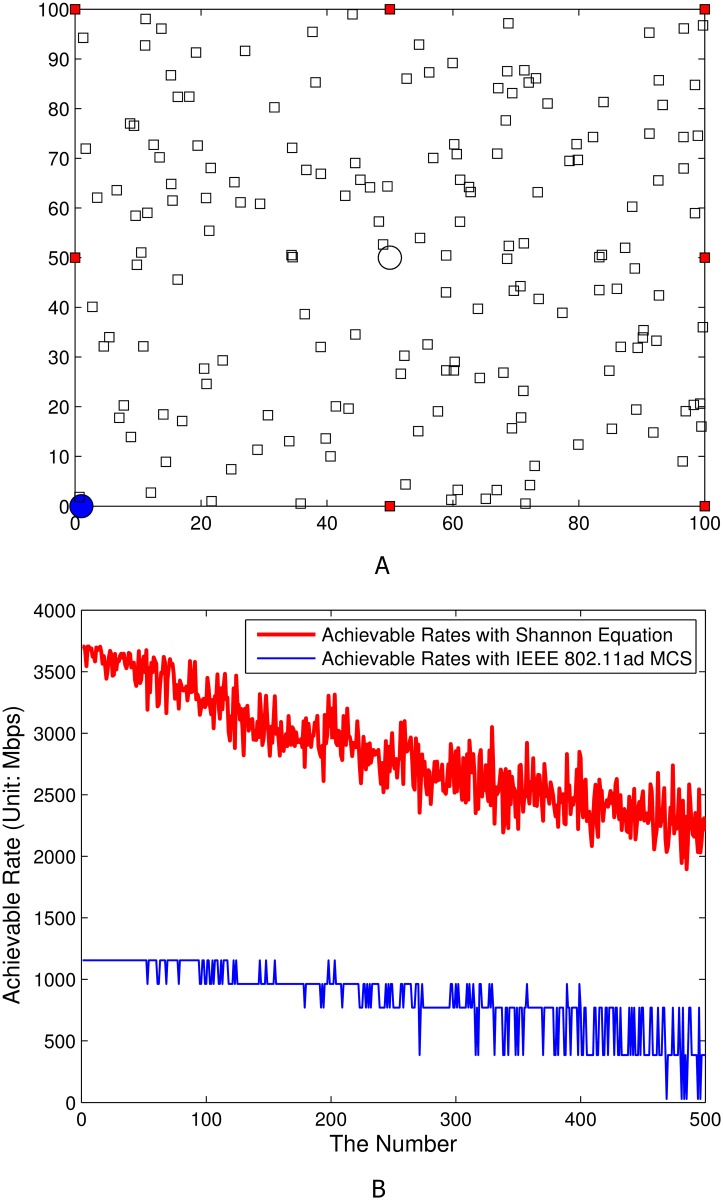
Simulation results with fairly deployed interfering CAPs. A) Network layout. B) Achievable rates depending on the number of deployed interfering SCDs (from 1 to 500).

**Fig 5:**
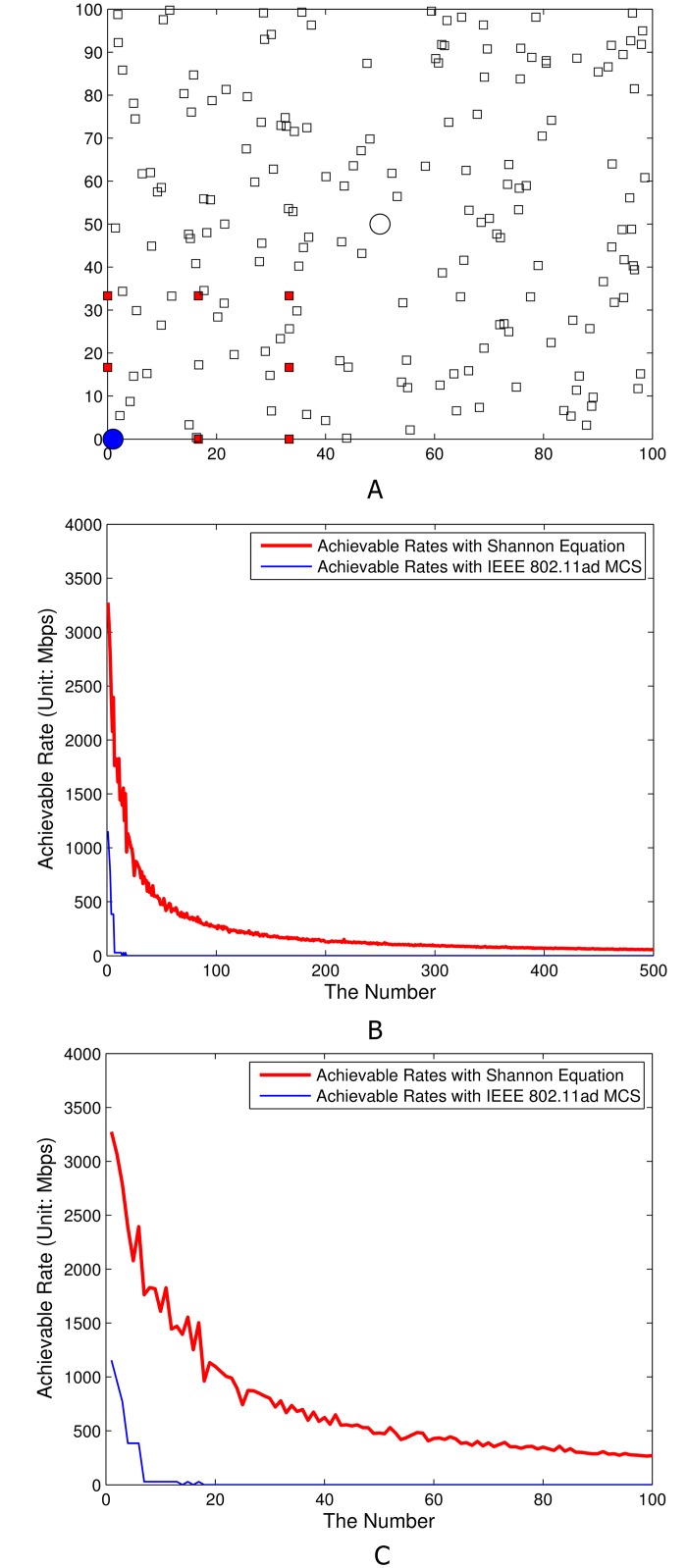
Simulation results with densely deployed interfering CAPs near a target CAP. A) Network layout. B) Achievable rates depending on the number of deployed interfering SCDs (from 1 to 500). C) Fig 5B where the number of SCDs is from 0 to 100.

The scale of network topology for this simulation study is 100 meters-by-100 meters; and the heights of CAPs and SCDs are assumed to be 6 meters and 1.5 meters. For this simulation study, the CAP and SCD of the main wireless link (i.e., the 60 GHz directional wireless link where SINR should be measured; and the CAP and the SCD are named as *target CAP* and *target SCD* in this simulation study) are located in (0, 0) and (50, 50) in the simulation network layout of 100 meters-by-100 meters. Then two separated deployments of interference components, i.e., (i) fair interfering CAPs distribution around borderlines (as illustrated in Fig 4A) and (ii) densely deployed interfering CAPs nearby the target CAP (as illustrated in Fig 5A). In Figs 4A and 5A, red boxes, white boxes, a blue circle, and a white circle stand for interfering CAPs, interfering SCDs, a target CAP, and a target SCD, respectively. Therefore, we measure the amount of interferences (occurred by the transmission from white boxes to their associated red boxes) toward the main link (i.e., the transmission from a white circle to its associated blue circle). The SINR from target SCD to its associated target CAP will be calculated when there exists interferences by the transmission from interfering SCDs to their associated target CAP while the interfering SCDs are aligned with their associated interfering CAPs. In terms of the considering association algorithm, we implemented a radio signal strength indicator (RSSI) based association algorithm.

Due to the randomness of the locations of interfering SCDs, the azimuth and elevation angle directions of antenna radiation patterns are also random. Therefore, Monte Carlo (MC) simulation was performed for evaluating the impacts of interference with 50 iteration. For each MC iteration, various numbers of SCDs are considered from 1 up to 500 for emulating large-scale visual big-data information uploading impacts. After performing 50 MC iterations with 500 SCDs, the simulation results are averaged over all deployments.

For the given two layouts in Figs 4A and 5A, two different achievable rate calculation procedures were performed, i.e., (i) achievable rate estimation with Shannon capacity equation and (ii) achievable rate estimation with IEEE 802.11ad MCS.

Based on these intensive simulation results, the corresponding facts can be observed:

- As presented Fig 4B, the upper bound of the achievable rate (i.e., the situation where interference sources are not existing) derived from Shannon capacity equation is around 3.65 Gbps. In this case, the achievable rate derived from IEEE 802.11ad MCS is around 1.12 Gbps (MCS level: MCS4).
- For the case where the interfering CAPs are deployed fairly around the borderline (i.e., red boxes in Fig 4), the achievable rate with Shannon capacity equation of main link (from target SCD (i.e., white circle) to target CAP (i.e., blue circle)) becomes below 3 Gbps when the number of interfering SCDs becomes more than 100. The tendency of performance degradation is linear. If the number of interfering SCDs is maximum (i.e., 500) the achievable rate with Shannon capacity equation of main link is near 1.9-2 Gbps. For the achievable rate estimation with IEEE 802.11ad MCS, the performance in terms of achievable rates eventually becomes below 27.5 Mbps (MCS level: MCS0) if the number of SCDs is more than near 460. If the number of SCDs is less than 460, the lower bound of the achievable rate with IEEE 802.11ad MCS is 385 Mbps (MCS level: MCS1).
- For the case where the interfering CAPs (i.e., red boxes in Fig 5) are densely deployed near target CAP (i.e., blue circle in Fig 5) as presented in Fig 5, the achievable rate with Shannon capacity equation of main link (from target SCD to target CAP) quickly drops. With only 18 deployed interfering SCDs, the performance drops to near 1 Gbps. The main reason of this extremely low performance is the transmit antenna gains from the transmit antennas of interfering SCDs. If interfering CAPs are deployed near target CAP, the transmit antennas of interfering SCDs will be aligned to the near location where the target CAP is located in (because interfering CAPs are near the target CAP and the interfering SCDs will be associated with one of interfering CAPs). Therefore, the transmit antenna gain of every single interference sources (i.e., $G_{\text{dBi}}^{i,\text{Tx}}\;(\phi^{*},\theta^{*})$ in Eq (15)) should be very large (i.e., every single interference impact is very high with high probability). This phenomena impacts on the performance degradation in SINR computation in Eq (11). For the achievable rate estimation with IEEE 802.11ad MCS, the achievable rate becomes 27.5 Mbps (MCS level: MCS0) if the number of SCDs is more than 7 as shown in Fig 5C. If the number of SCDs is more than 18, even MCS0 cannot be used, i.e., the data transmission is not possible with IEEE 802.11ad protocols as plotted in Fig 5C. In addition, the maximum achievable rate is 1.12 Gbps when the number of SCDs is only 1 (MCS level: MCS4).

## 5 Concluding Remarks

This paper computes and estimates the practical impacts of wireless interference on 60 GHz IEEE 802.11ad uplink access for large-scale visual big-data information uploading. For the computation of interference impacts, practical 60 GHz path-loss models and corresponding steerable antenna radiation patterns with Gaussian mainlobe profile are considered. Furthermore, this paper considers not only Shannon capacity based theoretical limits but also IEEE 802.11ad standard based practical modulation and coding scheme (MCS) set in order to estimate practical achievable data rates. On top of the given information, intensive numerical simulations with Monte Carlo iteration are performed with two different types of geometric settings. As discussed in the simulation results, the deployment of interference sources heavily impacts on the performance of uplink data transmission. Therefore, well-deployment of 60 GHz cloud access points is important in large-scale and dense wireless networks. This result is especially meaningful for the cloud access point placement applications where a lot of users and wireless equipments are densely deployed and simultaneously accessing the cloud access points.

## Supporting Information

S1 File Matlab source code. (ZIP)
