# Supplementary figures and images for: Achievable Rate Estimation of IEEE 802.11ad Visual Big-Data Uplink Access in Cloud-Enabled Surveillance Applications
Source: PLoS One. 2016 Dec 20;11(12):e0167447. doi: 10.1371/journal.pone.0167447 (PMC5173383; doi:10.1371/journal.pone.0167447)

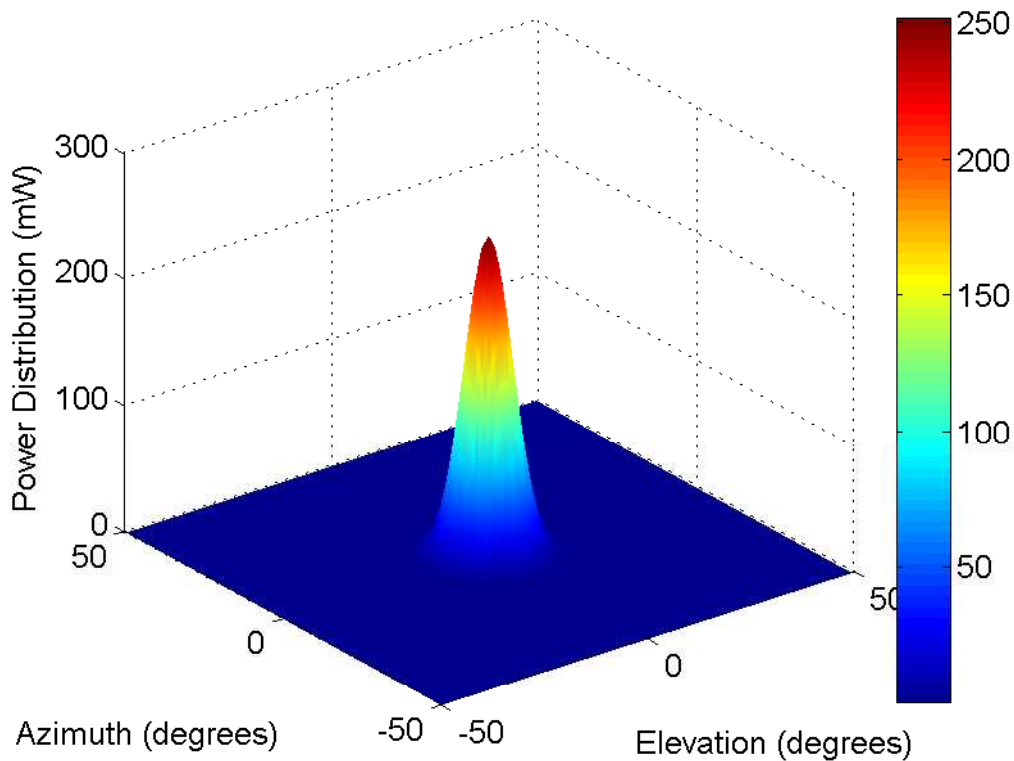

Supplement: S1 File — (ZIP) [file pone.0167447.s001.zip › antenna_3d_mW-eps-converted-to.pdf]

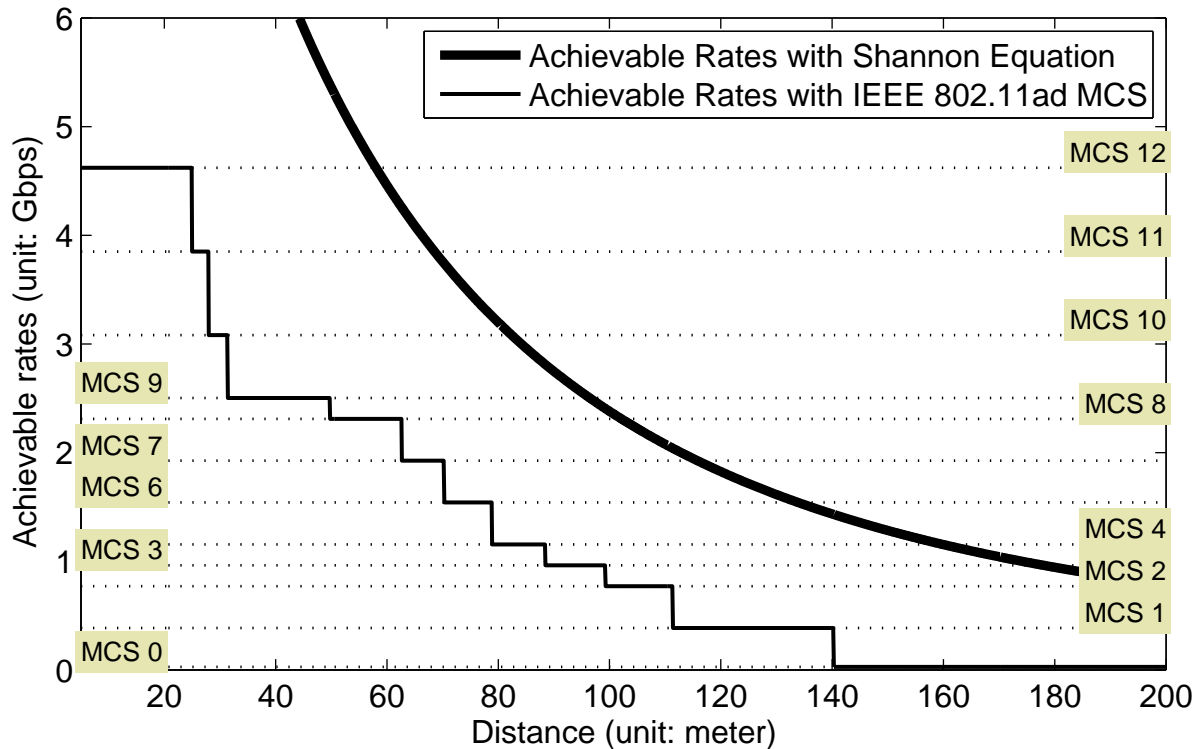

Supplement: S1 File — (ZIP) [file pone.0167447.s001.zip › snr-eps-converted-to.pdf]

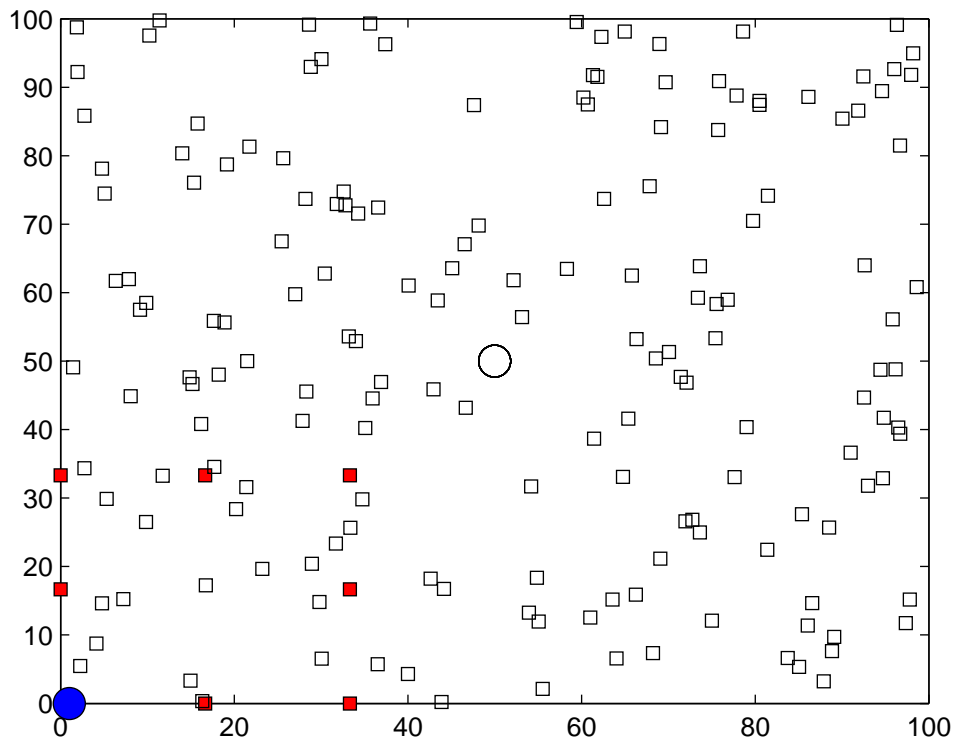

Supplement: S1 File — (ZIP) [file pone.0167447.s001.zip › dense-near/near-layout-eps-converted-to.pdf]

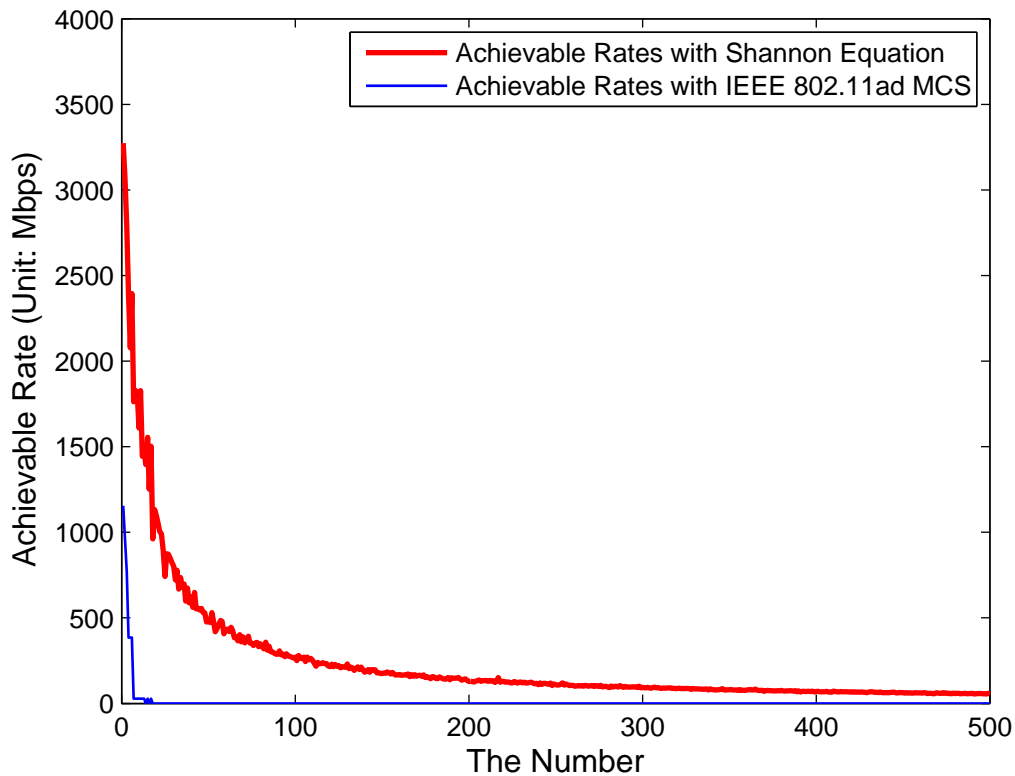

Supplement: S1 File — (ZIP) [file pone.0167447.s001.zip › dense-near/near-los-eps-converted-to.pdf]

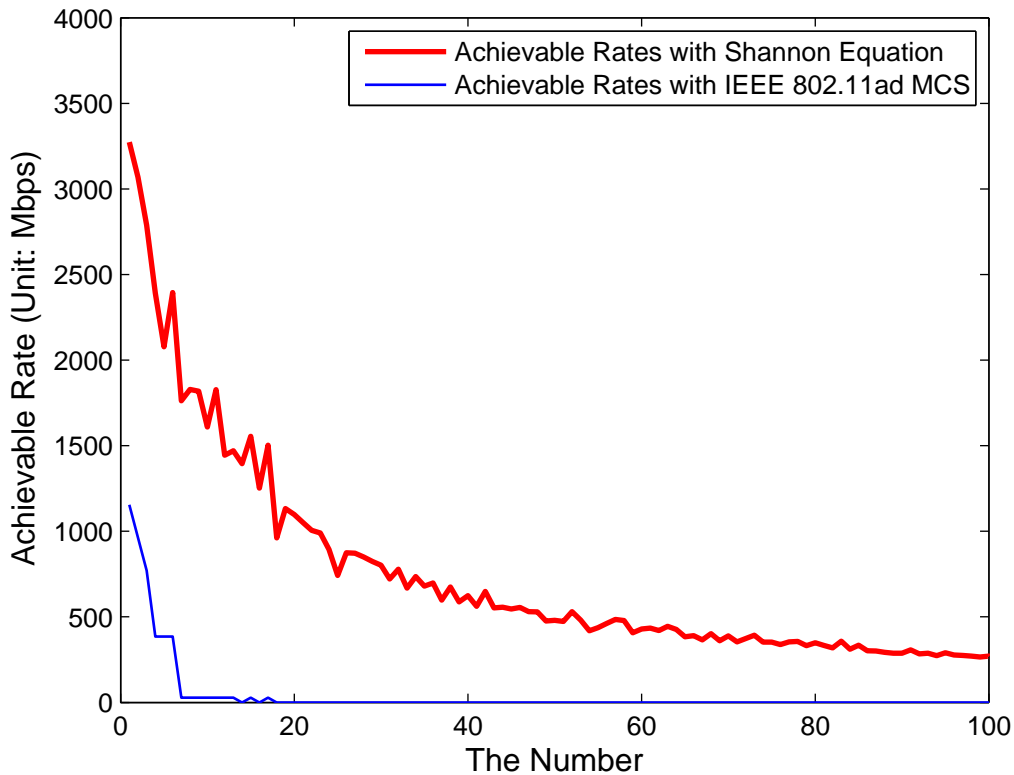

Supplement: S1 File — (ZIP) [file pone.0167447.s001.zip › dense-near/near-los2-eps-converted-to.pdf]

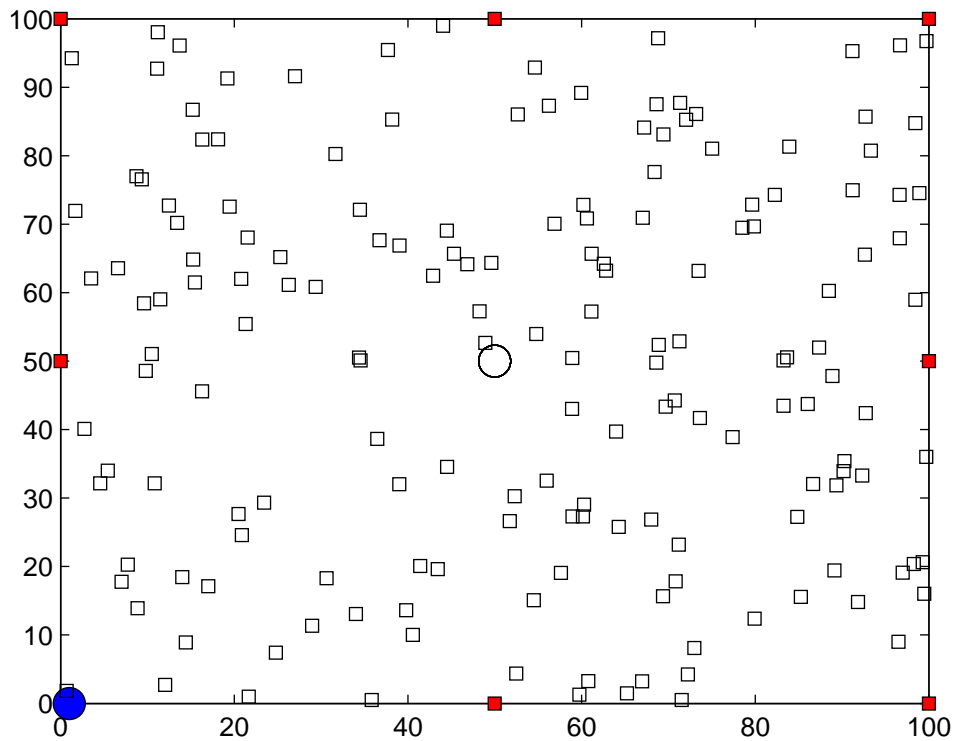

Supplement: S1 File — (ZIP) [file pone.0167447.s001.zip › fair/fair-layout-eps-converted-to.pdf]

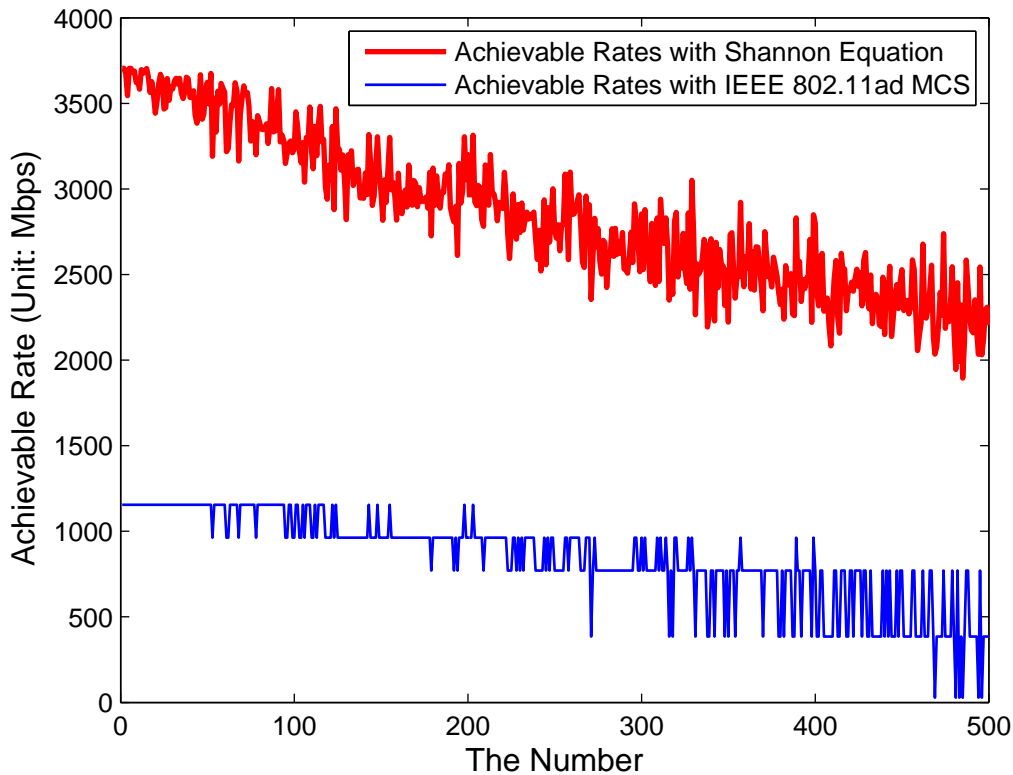

Supplement: S1 File — (ZIP) [file pone.0167447.s001.zip › fair/fair-los-eps-converted-to.pdf]
